# A misdiagnosed cause of early postoperative bowel obstruction

**DOI:** 10.1002/ccr3.2052

**Published:** 2019-02-17

**Authors:** Konstantinos Blouhos, Konstantinos A. Boulas, Aikaterini Paraskeva, Alexandros Triantafyllidis, Ioannis Kariotis, Anestis Hatzigeorgiadis

**Affiliations:** ^1^ Department of General Surgery General Hospital of Drama Drama Greece

**Keywords:** bowel obstruction, horseshoe kidney, mesenteric artery syndrome

## Abstract

In the setting of altered anatomy, diagnosis of superior mesenteric artery syndrome requires high clinical and imaging suspicion as the defined imaging criteria cannot be applied.

A 78‐year‐old male patient presented to our surgical department due to a recent history of abdominal distention, bilious vomiting, obstipation, and weight loss of 22 kg. Computed tomography (CT) depicted: (a) circumferential cecal wall thickening with fat infiltration and pericolic lymphadenopathy (Figure 1A); and (b) a horseshoe kidney with a giant simple cyst at the isthmus of the renal fusion (Figure 1B). Colonoscopy demonstrated a nearly obstructing cecal mass. Open right colectomy with complete mesocolic excision and central vascular ligation performed for a poorly differentiated cT4cN1‐2M0 cecal adenocarcinoma. Immediate postoperative period was characterized by continuation of bilious vomiting and obstipation. Nonoperative management of supposed early postoperative adhesive obstruction was initiated unsuccessfully. Open adhesiolysis performed on postoperative day 22 without recognition of a transition point. Despite relaparotomy, increased nasogastric drainage and bilious vomiting continued. The patient gradually died on postoperative day 42 due to intractable cachexia.

**Figure 1:**
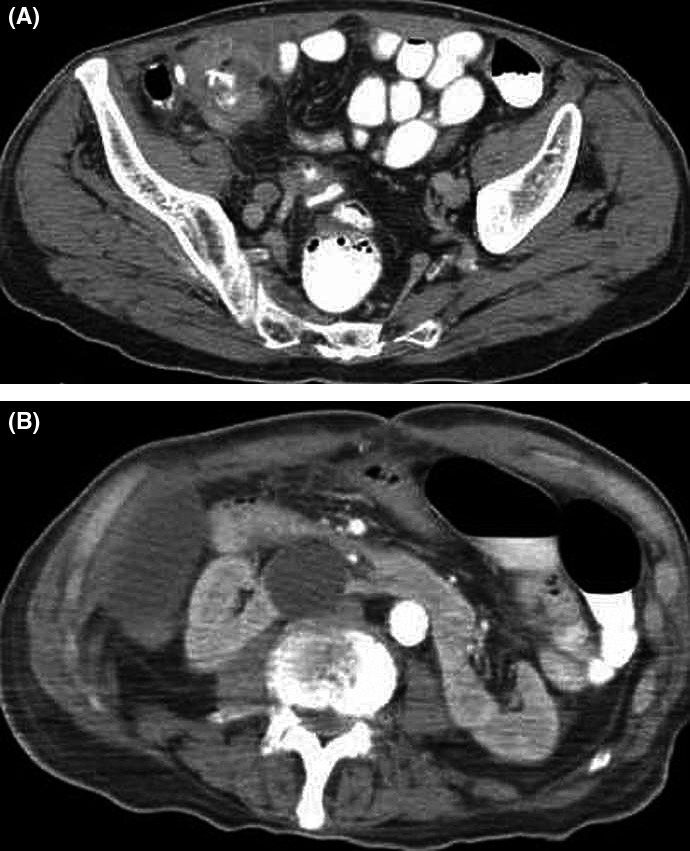
A, CT showed thickening of cecum wall with luminal narrowing. B, CT showed horseshoe kidney with a giant simple cyst at the isthmus of the renal fusion

## 1 QUIZ QUESTION: WHAT WAS MISDIAGNOSED?

**Figure 2:**
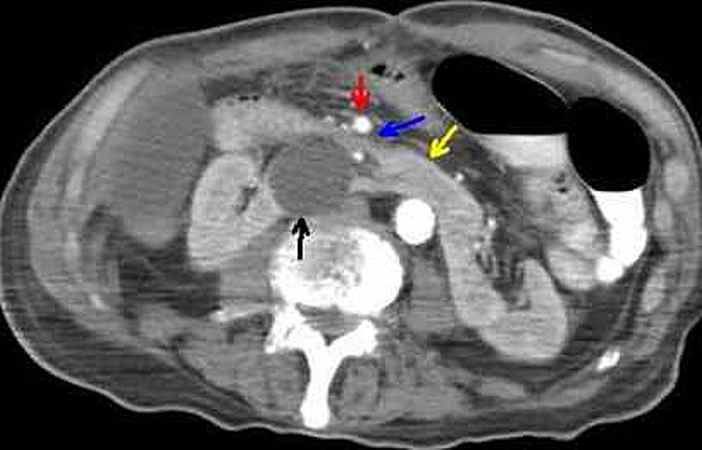
Review of CT images revealed duodenal dilation proximal to the level of the 3rd part (blue arrow) which was compressed by the close proximity of superior mesenteric artery (red arrow) to the renal cyst (black arrow) and isthmus (yellow arrow) of the horseshoe kidney. Aortomesenteric angle and distance had values above normal

Review of CT images enabled visualization of duodenal vascular compression. The 3rd part of duodenum was compressed by the close proximity of the superior mesenteric artery (SMA) to the renal cyst and isthmus, as the distance between SMA and renal cyst was 6 mm (Figure 2).^1^ In the present case, the defined imaging criteria for diagnosis of SMA syndrome (aortomesenteric angle of <22° and distance of <8‐10 mm) were not applied as both parameters had values above normal due to the presence of a horseshoe kidney which altered anatomic relationships between the aorta and SMA.^2^ Eventually, the patient suffered from a concurrent misdiagnosed chronic SMA syndrome. The sequence of events was as follows: Delayed diagnosis of colon cancer resulted in incomplete bowel obstruction, acute weight loss, and exacerbation of chronic SMA syndrome which finally dominated clinical presentation.

## 2 STATEMENT OF HUMAN AND ANIMAL RIGHTS

The present article does not contain any studies with human or animal subjects performed by any of the authors.

## CONFLICT OF INTEREST

The authors declare that they have no conflict of interests.

Informed consent: Informed consent was obtained from the patient.

## AUTHOR CONTRIBUTION

All authors equally accessed the data and contributed to the preparation of the manuscript. BK, BKA, and HA: were equally responsible for making and performing treatment decisions. HA: reviewed the manuscript for critical intellectual content and had the final approval.
